# Chebulinic acid suppresses porcine epidemic diarrhea virus infection by inhibiting viral entry and viral main protease

**DOI:** 10.3389/fcimb.2025.1531415

**Published:** 2025-03-18

**Authors:** Zhonghua Li, Yizhi Huang, Yi Zhang, Di Zhao, Lei Wang, Zhanchang Wang, Qunbing Hu, Ling Yang, Tao Wu, Yongqing Hou

**Affiliations:** ^1^ Hubei Key Laboratory of Animal Nutrition and Feed Science, Wuhan Polytechnic University, Wuhan, China; ^2^ Hubei Horwath Biotechnology Co., Ltd., Xianning, China; ^3^ Forestry and Fruit Tree Research Institute, Wuhan Academy of Agricultural Sciences, Wuhan, China

**Keywords:** porcine epidemic diarrhea virus, chebulinic acid, main protease inhibitor, attachment, penetration

## Abstract

Porcine epidemic diarrhea virus (PEDV) has resulted in significant economic losses in the global swine industry, making the development of effective therapeutic approaches a pressing need. In this study, we found that chebulinic acid significantly restrained PEDV replication in CCL-81 and LLC-PK1 cells, demonstrated by reductions in viral genome, viral protein, and titer. Molecular docking analysis made it clear that chebulinic acid might bind the key amino acids of binding pocket and the active center of PEDV main protease. Subsequent *in vitro* experiments confirmed the inhibitory effects of chebulinic acid on PEDV main protease, with an IC_50_ value of 61.53 ± 2.12 μM determined through a fluorescence resonance energy transfer (FRET) assay. Additional investigations demonstrated that chebulinic acid could inhibit the attachment and penetration processes of PEDV infection. Overall, our results provide experimental evidence supporting the inhibitory effects of chebulinic acid on PEDV infection by targeting viral entry and the inhibitory effects on main protease. The results of this study offer potential for creating new treatments for porcine epidemic diarrhea.

## 1 Introduction

Porcine epidemic diarrhea (PED) is one of the most significant viral diarrheal diseases affecting pigs, characterized by symptoms such as vomiting, watery diarrhea, dehydration, and high mortality rates in piglets. PED was initially identified in the United Kingdom in 1971 (Wood, 1977) and subsequently spread to various countries across Europe and Asia over the following decades (Sun et al., 2016). Since 2010, numerous countries across Asia, Europe, and America have witnessed new PED outbreaks, leading to significant damages in the global pig trade (Jung et al., 2020; Zhang et al., 2023).

The causative agent of this disease is the porcine epidemic diarrhea virus (PEDV), which belongs to the genus *Alphacoronavirus* in the Coronaviridae family (Pensaert and Bouck, 1978). The PEDV genome is composed of a single-stranded positive-sense RNA, featuring a minimum of seven open reading frames (ORF) (Lee, 2016). Among these, the five ORFs at the 3′ domain encode important structural proteins such as the spike protein (S), the envelope protein (E), the membrane protein (M), and the nucleocapsid protein (N), along with a nonstructural protein known as ORF3 (Peng et al., 2023). These proteins play vital roles in forming the viral envelope and nucleocapsid, which give the virion its distinct structure and size range of 95–190 nm. Additionally, the two ORFs at the 5′ domain, ORF1a and ORF1b, account for about two-thirds of the PEDV genome and encode polyproteins that are crucial for viral replication (Wilde et al., 2017; Chen et al., 2020). The cleavage of these polyproteins by the coronavirus non-structural protein 5 (nsp5), also known as main protease, is essential for the production of intermediate and mature nonstructural proteins (Zhou et al., 2024). Main protease has long been a target of drug development against coronaviruses due to the key role it plays in viral replication, making it an important target for anti-coronavirus therapy (Xiong et al., 2021; Jiang et al., 2023).

*Terminalia chebula* Retz. (*T. chebula* Retz.) belongs to the *Combretaceae* family and is one of the most frequently utilized medicinal plants in various Tibetan traditional medicinal prescriptions for treating a range of diseases (Longhe et al., 2020). The *T. chebula* extract contains a diverse array of bioactive compounds, including tannins, phenolic acids, and flavonoids, which confer multiple beneficial properties, including anti-diabetic, anti-inflammatory, antioxidant, hepatoprotective, neuroprotective, and gastroprotective effects (Nigam et al., 2020). Chebulinic acid is a notable tannin-like molecule found in *T. chebula* extract. To date, some biological functions of chebulinic acid have been identified. It has demonstrated antioxidant (Sharma et al., 2023), antibacterial (Wahi et al., 2015), and anti-apoptotic (Chhabra et al., 2017) properties. Recently, chebulinic acid has gained recognition for its inhibitory effects on cancer (Sharma et al., 2020) and adipogenesis (Kim and Ahn, 2022). Importantly, accumulating evidence suggest that chebulinic acid exhibits an inhibitory activity against viral infections, including anti-herpes simplex virus type 2 (HSV2) (Kesharwani et al., 2017), anti-SARS-CoV (Ojha et al., 2023), anti-influenza virus (Li et al., 2020a), anti-dengue virus (DENV), and anti-chikungunya virus (CHIV) (Thomas et al., 2022). However, there is limited knowledge regarding the antiviral effects of chebulinic acid on anti-PEDV.

In this study, we discovered that chebulinic acid showed an anti-PEDV effect by suppressing the activity of PEDV main protease and viral entry. Given its promising anti-PEDV effects, we aim to explore chebulinic acid’s potential as a novel drug for addressing the challenges posed by PED.

## 2 Materials and methods

### 2.1 PEDV strains, cell lines, chebulinic acid, and antibody

The PEDV YN13 strain, a member of GIIb strains, was isolated and purified from the jejunum of a diarrheal pig in 2015. The DR13-dORF3/GFP strain was a lab-engineered PEDV strain which was obtained by replacing the ORF3 gene of DR13 strain (one of the GIa strains) with the green fluorescent protein (GFP) gene by a PEDV infectious clone. The CCL-81 cell line (renal cells from African green monkey) and the LLC-PK1 cell line (porcine kidney epithelial cells) were stored in our laboratory. Chebulinic acid (>99%) was purchased from MedChemExpress (Shanghai, China). The mouse monoclonal antibody (MAb) for recognizing the S protein of PEDV was prepared from the special hybridoma which was established by our laboratory.

### 2.2 Cytotoxicity assay

This experiment began by seeding the cells into a 96-well plate, followed by incubation at 37°C with 5% CO_2_ until a full monolayer developed. Subsequently, the cells underwent incubation with chebulinic acid of various concentrations for another 48 h. Following three washes with PBS, 100 μL of a 10% CCK-8 solution (CCK-8 to DMEM ratio 1:9) was added to each well. In addition, a negative control group was set up by adding 10% CCK-8 solution in the wells without cells. The optical density values at 450 nm (OD450) of each well were analyzed using the spectraMax i3x platform after an incubation period of 2 h. Cell viability was calculated using the formula: cell viability = [A - B] / [C - B]. In this equation, A represents the OD450 of the experimental wells (chebulinic-acid-treated cells), B represents the OD450 of the mock wells (the wells without cells), and C represents the OD450 of the control group (cells without chebulinic acid).

### 2.3 Real-time quantitative reverse transcription PCR

For the YN13 strain, CCL-81 or LLC-PK1 cells were seeded in 12-well culture plates. The cells were infected with PEDV in the presence of chebulinic acid (0, 12.5, 25, or 50 μM) for a duration of 24 h when grown to approximately 90% confluence. In addition, during the infection with the YN13 strain, it is essential to add a culture medium containing 8 μg/mL of trypsin, with the infection multiplicity (MOI) set at 0.001. For the DR13-dORF3/GFP strain, the treatments were similar to the procedures for the YN13 strain except that trypsin was not required and the duration was 36 h. Three replicates were set up for each concentration, with one well per replicate. The methods for RNA extraction, RT-PCR for producing cDNA, and the real-time RT-PCR were referred to our previous study (Li et al., 2024). Real-time RT-PCR information of primers is provided in **Table 1**.

**Table 1:** Information of primers for the real-time RT-PCR.

Gene		Sequence
*HPRT1* (CCL-81)	Forward	5′-AACCTTGCTTTCCTTGGTCA-3′
	Reverse	5′-TCAAGGGCATAGCCTACCAC-3′
*RPL19* (LLC-PK1)	Forward	5′-AACTCCCGTCAGCAGATCC-3′
	Reverse	5′-AGTACCCTTCCGCTTACCG-3′
*PEDV M*	Forword	5′-TCCCGTTGATGAGGTGAT-3′
	Reverse	5′-AGGATGCTGAAAGCGAAAA-3′

Porcine ribosomal protein L19 (RPL19) and homo sapiens hypoxanthine phosphoribosyltransferase 1 (HPRT1) were used as the housekeeping gene for LLC-PK1 and CCL-81 cells, respectively.

### 2.4 Indirect immunofluorescence assay

The methods employed for PEDV infection and chebulinic acid treatment are detailed in Section 2.3. For IFA, the cells were first washed with PBS, followed by fixation with 4% paraformaldehyde at 37°C for a duration of 10 min. The cells were washed thrice with PBS, and subsequent to discarding the fixative, they were treated with pre-chilled methanol at -20°C for 15 min. After washing with PBS, 5% bovine serum albumin (BSA) solution (PBS as the solvent) was used for blocking non-specific binding sites. After blocking, the cells were incubated with the anti-PEDV S protein MAb at 37°C for 1 h. The cells were washed with PBS to completely remove the primary antibody, and then the fluorescently labeled second antibody was added to each well for 45 min at 37°C under light protection. The cell nuclei were stained by incubating with DAPI solution at 37°C for 5–10 min. After washing with PBS, the cells were observed, and pictures were acquired using an inverted fluorescence microscopy imaging system (Olympus, Tokyo, Japan).

### 2.5 Fluorescence observation of the DR13-dORF3/GFP-infected cells

The DR13-dORF3/GFP strain (MOI = 0.001) was used to infect the CCL-81 monolayer (90% confluence). Meanwhile, chebulinic acid of different concentrations was added for 36 h. The cells were washed with PBS, followed by incubating with Hoechst 33258 solution to label the cell nuclei under light protection. The cells were observed, and pictures were acquired using an inverted fluorescence microscopy imaging system (Olympus, Tokyo, Japan).

### 2.6 Tissue culture infectious dose 50 assay

In order to access the virus titers, the cells were treated as described in Section 2.3. Following three cycles of freezing and thawing process, the PEDV whole culture (comprising culture medium and cells) was centrifuged to yield supernatant samples for subsequent viral titer determination. The samples for the TCID_50_ assay were serially diluted in a 10-fold manner before being added to the CCL-81 cell monolayers in a 96-well plate. YN13 strain samples were attenuated using DMEM containing 8 μg/mL of trypsin, while the samples of DR13-dORF3/GFP strain were diluted using DMEM without trypsin. Eight repetitions are required for each dilution. The number of wells infected with PEDV was determined daily by microscopic examination over the subsequent 3 days. For the samples of YN13 strain, the wells exhibiting multinucleate cells, also known as syncytium formation, were classified as positive. The samples of DR13-dORF3/GFP strain, the wells displaying green fluorescence upon excitation with blue light, were identified as positive. Viral titration was determined in accordance with a previously established methodology (Reed and Muench, 1938).

### 2.7 Docking of the PEDV main protease and chebulinic acid

The structures of chebulinic acid and PEDV main protease (PDB: 5HYO) were downloaded from ZINC15 and RCSB PDB data bank, respectively. The autodock vina software was used to create the binding models of PEDV main protease and chebulinic acid with 74 × 68 × 110 grid points (center: *x* = 35.786, *y* = 13.412, *z* = -30.837; spacing = 1.000) covering all the amino acid sites of PEDV main protease. The parameters for this docking were configured as follows: a population size of 150, a maximum energy evaluation limit of 5,000,000, a maximum number of generations set to 27,000, a mutation rate of 0.02, and a crossover rate of 0.8. A total of 10 docked conformations were produced, and the one with the minimum binding energy was selected to represent the binding details of chebulinic acid with the PEDV main protease.

### 2.8 Expression and purification of PEDV main protease

The PEDV nsp5 gene of the PEDV YN13 strain was cloned into the pET-28a (+) vector to create a recombinant vector (pET-28a-NSP5) for the expression of the PEDV main protease. The recombinant bacteria expressing the PEDV main protease was constructed by transforming *E. coli BL21* (*DE3*) with the Pet-28a-NSP5 vector. The bacteria were cultivated in a double-layer shaking incubator (Zhicheng, Shanghai, China) at 37°C with a shaking speed of 180 rpm until the optical density at 600 nm (OD600) reached 0.6–0.8. Subsequently, a dosage of 0.8 mM isopropyl β-D-thiogalactoside was administered, and the culture was continued at 37°C to induce the expression of the PEDV main protease. The bacteria were harvested and resuspended in ice-pre-cooled PBS and disrupted using a high-pressure homogenizer (Litu, Shanghai, China) at 4°C–6°C. After having been filtered with a 0.45-μM filter (BKMAM, Changsha, China), the supernatant was loaded onto a Ni-sepharose column (Nuptec, Hangzhou, China). Finally, the PEDV main protease with His-tag at both N and C terminals was eluted and then desalted and concentrated by ultrafiltration.

### 2.9 Fluorescence resonance energy transfer assays

Based on previous studies (Ye et al., 2016, 2020), a peptide substrate with a conserved cleavage site (YNSTLQ↓AGLRKM) was designed to detect the cleavage activity of the PEDV main protease and synthesized by Genscript Biotech Corporation (Nanjing, China). The peptide substrate is provided in **Table 2**. The enzyme reaction system for testing PEDV main protease activity, with a total volume of 100 μL, included 20 mM Tris/HCl buffer, 10 μM peptide substrate, and various concentrations of the purified PEDV main protease. Fluorescence intensity was measured at 5-min intervals over a period of 60 min, using an excitation wavelength of 340 nm and an emission wavelength of 485 nm, on the SpectraMax i3x platform. The reaction systems (100 μL) for analyzing the inhibitory effects of chebulinic acid on PEDV main protease included 10 μM peptide substrate, 1 μM main protease, various concentrations of chebulinic acid, and 20 mM Tris/HCl buffer. Pretreatment involved incubating chebulinic acid with the main protease for 30 min at 37°C, followed by the addition of the substrate to this mixture. A control group lacking chebulinic acid was also prepared. Four replicates were set up for each concentration, with one well per replicate. The fluorescence emissions were tracked at excitation 340 nm and emission 485 nm using the SpectraMax i3x system.

**Table 2:** Information on peptide substrate for FRET.

Name	Sequence
FRET substrate	Dabcyl-YNSTLQ↓AGLRKM-E-Edans

The formula used to determine the suppression ratio of chebulinic acid on the PEDV main protease is as follows: suppression ratio (%) = [1 - (fluorescence intensity of experimental group at 60 min - fluorescence intensity of experimental group at 0 min) / (fluorescence intensity of control group at 60 min - fluorescence intensity of control group at 0 min)] × 100.

### 2.10 Time-of-addition assays

The CCL-81 in 12-well plates were infected with YN13 or DR13-dORF3/GFP strain (MOI = 0.001) in the presence of 50 μM chebulinic acid at different time points, namely: (a) incubation of chebulinic acid and PEDV for 2 h before infection, (b) 2 h before infection (-2–24 h), (c) simultaneously with PEDV infection (0–24 h), (c) 2 h post-infection (2–24 h), and (d) 4 h post-infection (4-24 h). Three replicates were set up for each concentration, with one well per replicate. RNA extraction was performed at 24 h post-infection, followed by real-time RT-PCR analysis conducted in accordance with Section 2.3.

### 2.11 Adsorption and penetration assay of PEDV infection

Following a previous study, the attachment and penetration assays were carried out (Hong et al., 2014) with some minor modifications. For the attachment assay, CCL-81 monolayers in 12-well plates were pre-chilled at 4°C for 30 min and infected with YN13 strain or DR13-dORF3/GFP strain (MOI = 0.001) for 2 h at 4°C, and different concentrations of chebulinic acid were added. After rinsing (three washes) with pre-cooled PBS, the cells were harvested and subjected to detect the PEDV M gene through real-time RT-PCR. For the penetration assay, CCL-81 monolayers in 12-well plates were pre-chilled at 4°C for 30 min and infected with the YN13 or DR13-dORF3/GFP strain (MOI = 0.001) for 2 h at 4°C. Three replicates were set up for each concentration, with one well per replicate. The cells were rinsed three times with pre-cooled PBS. Viral penetration proceeded at 37°C in the presence of chebulinic acid of different concentrations. After 1 h of incubation, the cells were washed with PBS and replenished with a maintenance cover layer (DMEM with 8 μg/mL of trypsin for YN13; DMEM for DR13-dORF3/GFP). After an incubation period of 24 h, the cells were harvested and subjected to detect the PEDV M gene thorough real-time RT-PCR.

### 2.12 Statistical analysis

Green fluorescence intensity was analyzed using ImageJ software. SPSS 17.0 software was used to calculate the half maximal inhibitory concentration (IC_50_). Every experiment was carried out three times, and the results were exhibited in the form of mean values ± standard deviation (SD). Student’s *t*-test was performed to calculate the statistical significance. *P* > 0.05 means no significant difference compared with the control group and represented as ns. *p* < 0.05 was regarded to denote a significant difference compared with the control group and represented as *; *p* < 0.01 was considered to denote a significant difference compared with the control group and represented as **; and *p* < 0.001 was considered to denote an extremely significant difference compared with the control group and represented as ***.

## 3 Result

### 3.1 Toxicity of chebulinic acid on CCL-81 and LLC-PK1 *c*ells

**Figure 1:**
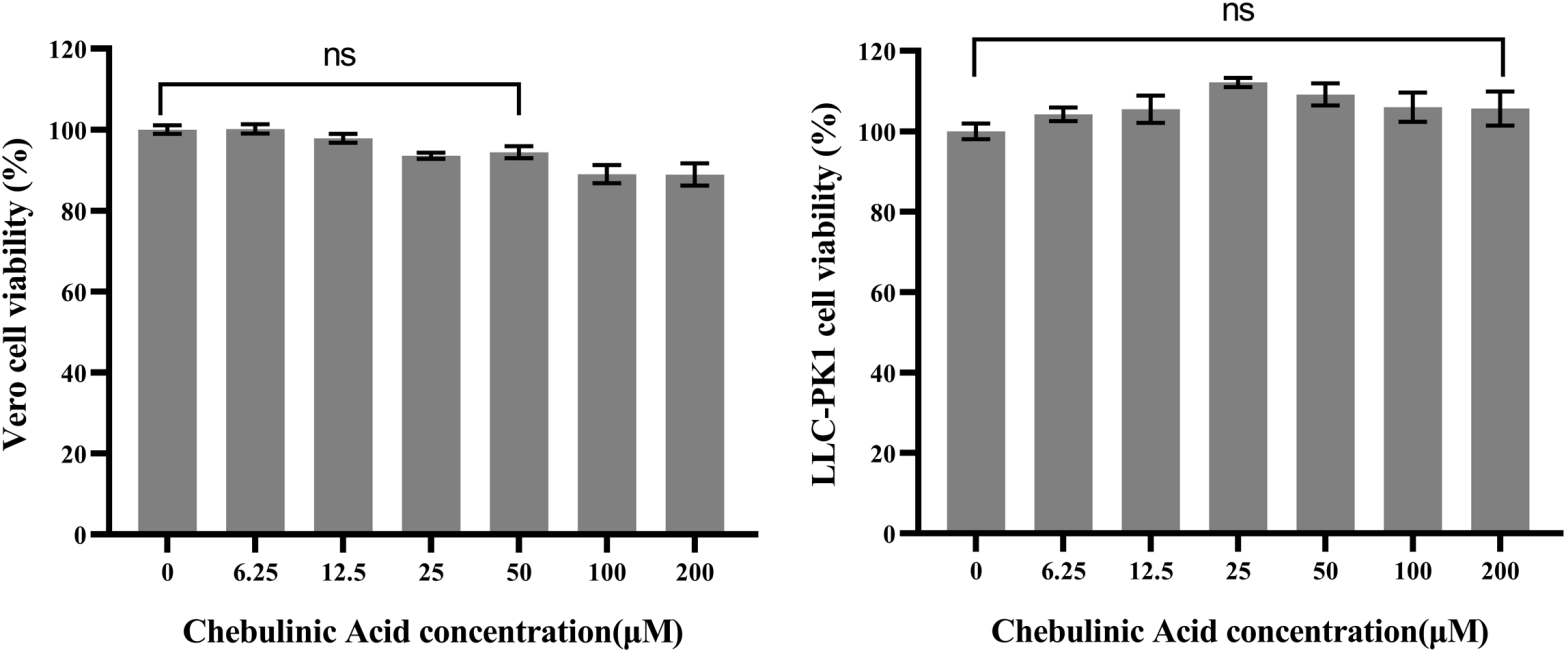
Cytotoxicity of chebulinic acid on CCL-81 or LLC-PK1 cells. The control group represents 100% cell viability. The cell viability of the other groups were normalized to this control group. *P* > 0.05 means no significant difference compared with the control group and represented as ns.

The viability of both CCL-81 and LLC-PK1 cells was assessed after being exposed to a range of chebulinic acid concentrations, with the aim of determining the safe concentration range for use in subsequent anti-PEDV studies. Our results demonstrated that there were no significant differences between the cell viability of the control group and the chebulinic-acid-treated groups at concentrations not more than 50 µM for both CCL-81 and LLC-PK1 cells (**Figure 1**).

### 3.2 Chebulinic acid inhibit PEDV infection in CCL-81 cells

**Figure 2:**
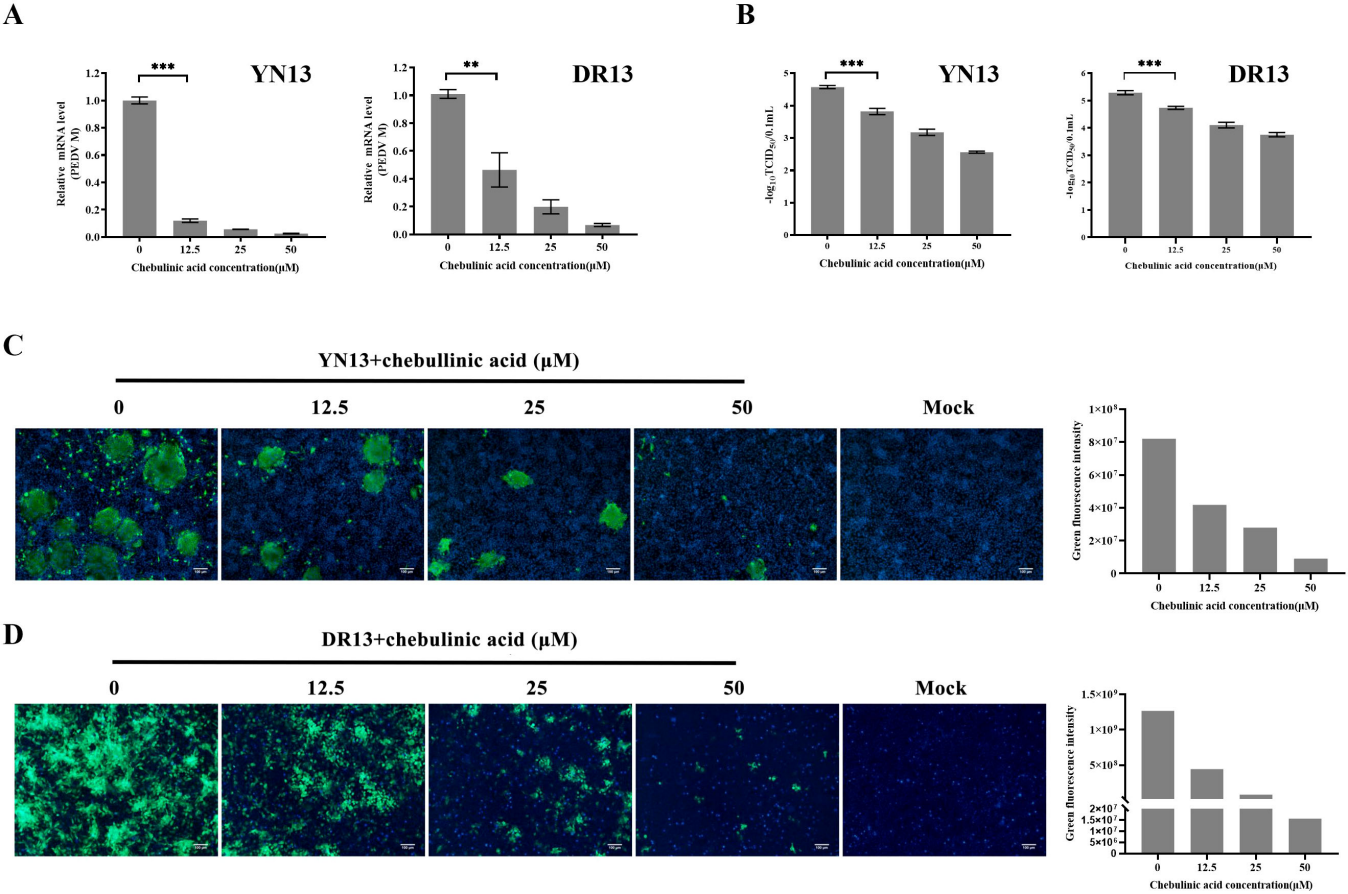
Anti-PEDV activity of chebulinic acid in CCL-81 cells. **(A)** The inhibition of chebulinic acid on PEDV M gene in PEDV infected CCL-81 cells. **(B)** The inhibition of chebulinic acid on PEDV titer. **(C)** The inhibition of chebulinic acid on the PEDV S protein expression in the YN13 strain infected CCL-81 cells. **(D)** The inhibition of chebulinic acid on the expression of GFP in the DR13-dORF3/GFP strain infected CCL-81 cells.

The antiviral effects of chebulinic acid against YN13 and DR13-dORF3/GFP strains were firstly investigated by using real-time RT-PCR. As shown in **Figure 2A**, chebulinic acid exhibited a dose-dependent inhibitory effect on PEDV M gene expression in CCL-81 cells infected with both PEDV strains. The effects of chebulinic acid against PEDV in CCL-81 cells were confirmed using a TCID_50_ assay. As illustrated in **Figure 2B**, significant reductions in the titers of both of these two PEDV strains were noted following the application of chebulinic acid. Additionally, IFA was also applied to demonstrate the suppressive effect of chebulinic acid on the number of YN13-strain-infected CCL-81 cells. The IFA results indicated a dose-dependent decrease in the quantity of viral infected cells following treatment with chebulinic acid. Moreover, the group treated with chebulinic acid demonstrated a reduction in both the size and quantity of syncytia, which were the typical cytopathic effects associated with the YN13-strain-infected cells (**Figure 2C**). Moreover, consistent with the observations from IFA results, chebulinic acid could decrease the GFP expression in DR13-dORF3/GFP-strain-infected CCL-81 cells (**Figure 2D**).

### 3.3 Chebulinic acid inhibit*s* PEDV infection in LLC-PK1 cells

**Figure 3:**
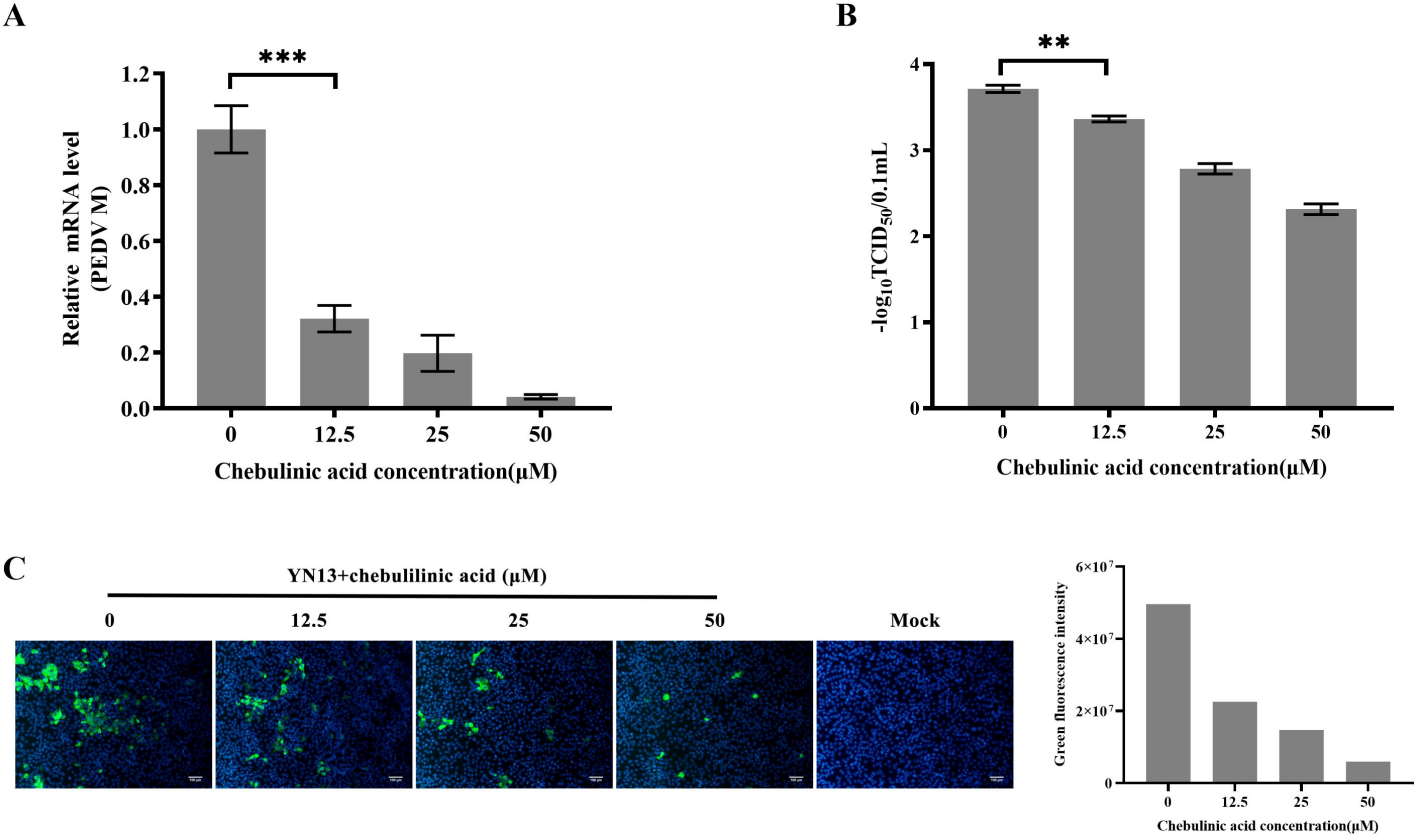
Anti-PEDV activity of chebulinic acid in LLC-PK1 cells. **(A)** Inhibition of chebulinic acid on PEDV M gene in PEDV-infected LLC-PK1 cells. **(B)** Inhibition of chebulinic acid on PEDV titer. **(C)** Inhibition of chebulinic acid on the PEDV S protein expression in the YN13-strain-infected LLC-PK1 cells.

A different cell line, LLC-PK1, was selected to further validate the inhibition of chebulinic acid on PEDV infection. The results observed in LLC-PK1 cells were consistent with those found in CCL-81 cells. Chebulinic acid significantly inhibited the proliferation of viral genome and reduced both the viral titers and the number of infected cells (**Figure 3**). According to all of the abovementioned results, it can be concluded that chebulinic acid is an effective inhibitor of PEDV infection.

### 3.4 Chebulinic acid may bind to the active site of PEDV main protease

**Figure 4:**
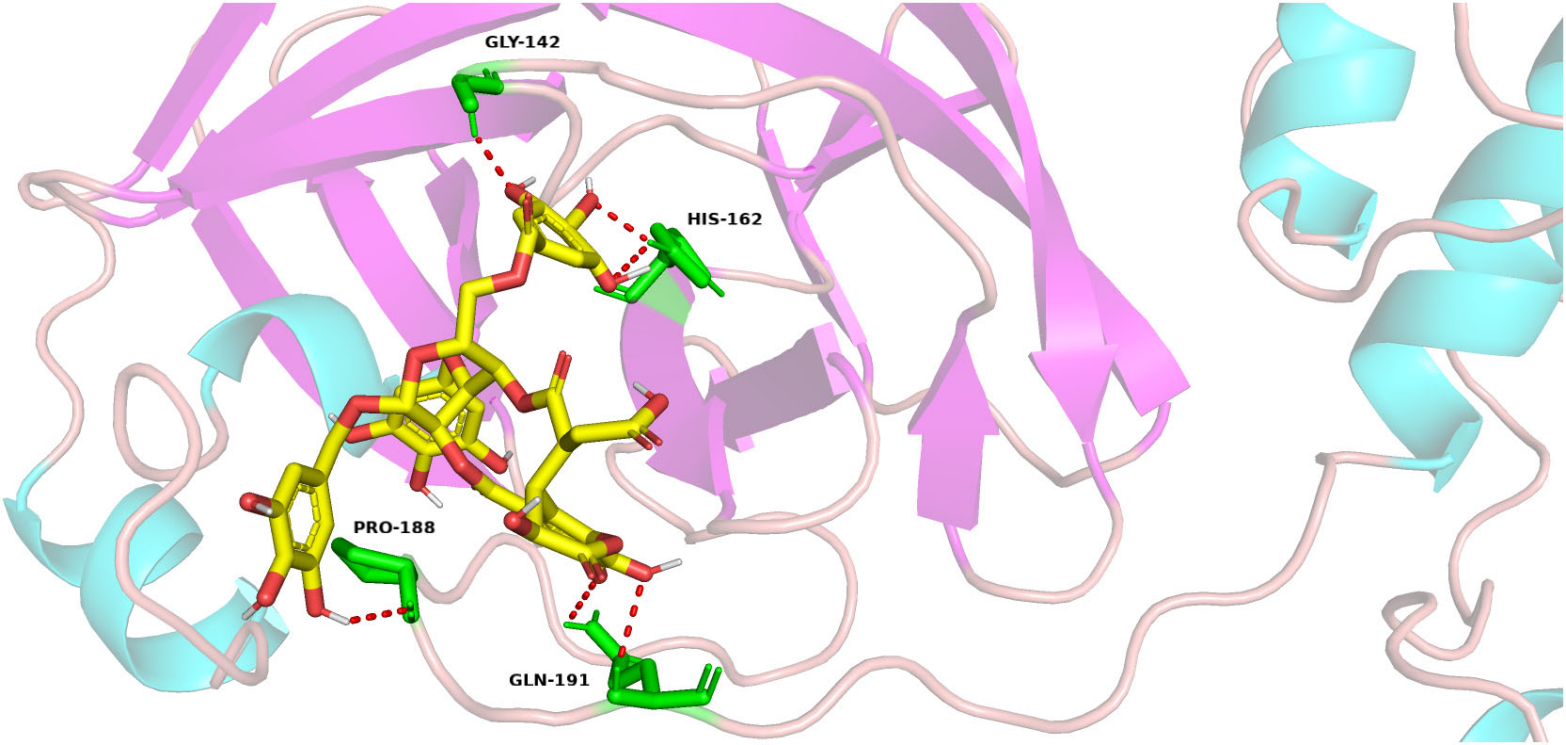
Docking of chebulinic acid with PEDV main protease. In the depicted structure, chebulinic acid is illustrated using sticks, with yellow indicating carbon atoms, red for oxygen atoms, and white for hydrogen atoms. The amino acid residues that interact with chebulinic acid are highlighted as green sticks, demonstrating the specific areas of contact within the enzyme. The hydrogen bond interactions between chebulinic acid and PEDV main protease are illustrated with red dashed lines, emphasizing the significance of these interactions in stabilizing the binding complex.

Chebulinic acid has been identified as a compound capable of binding to the main protease of SARS-CoV-2. This discovery prompted a subsequent docking analysis to investigate the interaction between chebulinic acid and the PEDV main protease. The analysis revealed that chebulinic acid interacts with several key amino acids within the active site of PEDV main protease, including Gly142, His162, Pro188, and Gln191, as illustrated in **Figure 4**. Consequently, these interactions suggest that chebulinic acid may effectively hinder the binding process between PEDV main protease and its substrates, thereby leading to the inhibition of PEDV infection.

### 3.5 Chebulinic acid inhibit*s* PEDV main protease enzymatic activity

**Figure 5:**
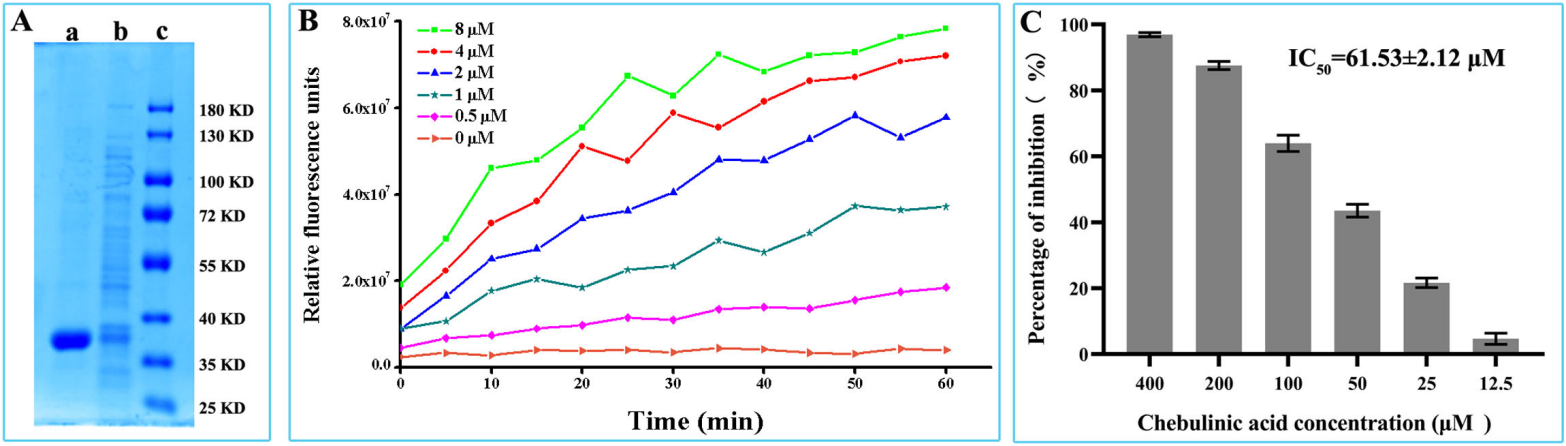
Inhibitory effect of chebulinic acid on PEDV main protease. **(A)** Expression and purification of PEDV main protease. Lane a, PEDV main protease purified by affinity chromatography; lane b, supernatant of the recombinant bacteria has been homogenized at high pressure; lane c, protein marker. **(B)** Enzyme activity assay of the purified PEDV main protease. **(C)** Inhibition of chebulinic acid on PEDV main protease activity.

The PEDV-main-protease-expressed vectors were constructed by cloning PEDV NSP5 gene into pet28a (+) vectors and then transfected into *BL21* for the prokaryotic expression of the PEDV main protease. As shown in **Figure 5A**, PEDV main protease could be expressed in a soluble form and purified by affinity chromatography. To detect the enzymatic activity of the purified protein, a FRET substrate was designed and synthesized according to the conserved cleavage site within PEDV polyproteins specifically recognized by PEDV main protease. According to the results of the FRET assay, the fluorescence value increased with increasing chebulinic acid concentrations, demonstrating that the purified PEDV main protease could effectively cleave its FRET peptide substrate (**Figure 5B**). These findings suggest that this method can be applied to access the cleavage activity of PEDV main protease and to test the inhibitory effect of chebulinic acid on PEDV main protease. To evaluate its inhibitory effect on PEDV main protease, different concentrations of chebulinic acid were added to the FRET assay. As illustrated in **Figure 5C**, chebulinic acid demonstrated a dose-dependent inhibitory effect on PEDV main protease cleavage activity with IC_50_ of 61.53 ± 2.12 μM.

### 3.6 Chebulinic acid inhibit*s* PEDV entry step

**Figure 6:**
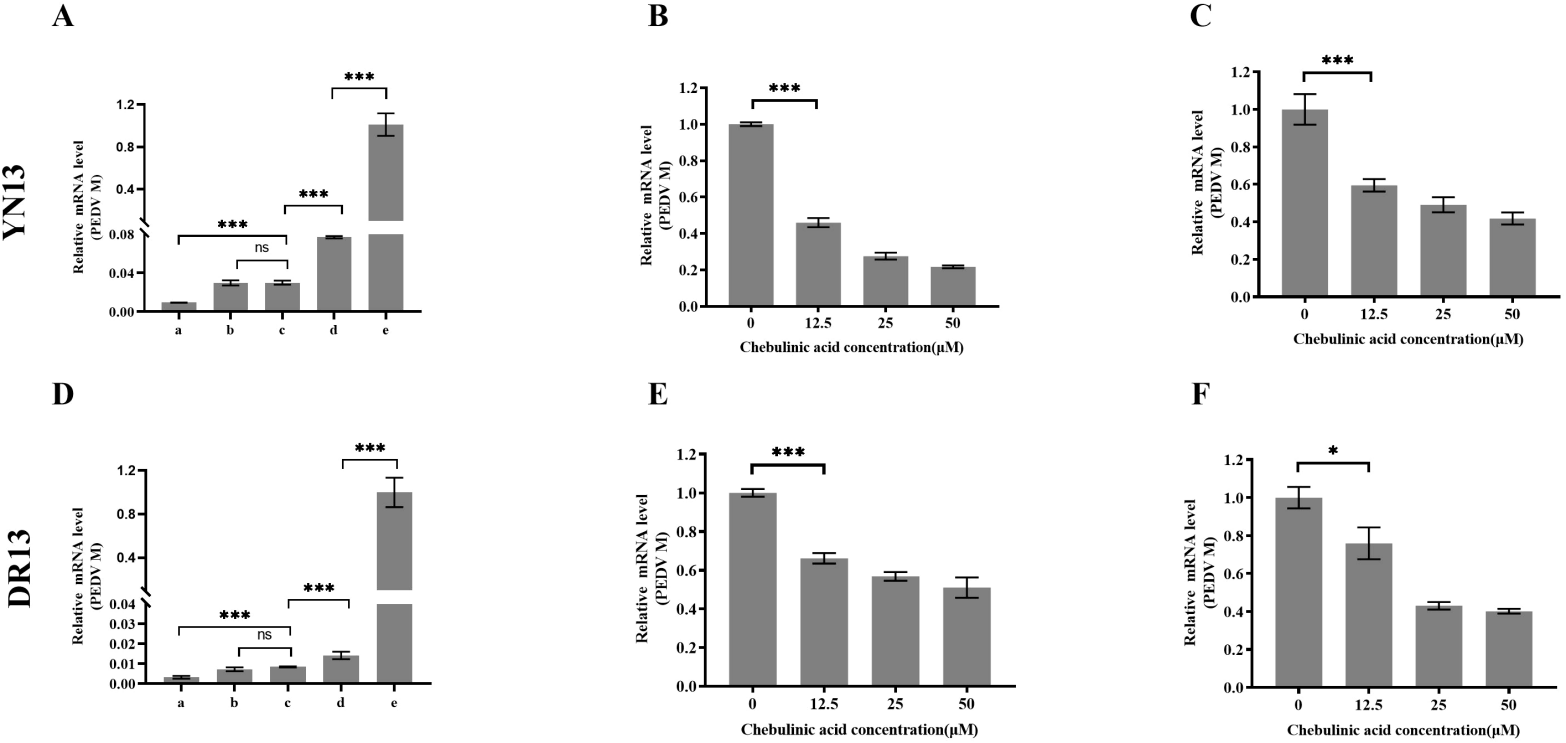
Inhibition of chebulinic acid on the entry step of PEDV infection. Time-of-addition effect of chebulinic acid on YN13 **(A)** and DR13-dORF3/GFP **(D)** proliferation. A total of 50 μM chebulinic acid was introduced to the culture medium at different stages of PEDV infection: a—incubation of chebulinic acid and PEDV for 2 h before infection; b—2 h before infection (-2–24 h); c—simultaneously with PEDV infection (0–24 h); d—2 h post-infection (2–24 h); e—PEDV infected in the absence of chebulinic acid. Inhibition of chebulinic acid on the attachment of YN13 **(B)** and DR13-dORF3/GFP **(E)** to CCL-81 cells. Inhibition of chebulinic acid on the penetration of YN13 **(C)** and DR13-dORF3/GFP **(F)** into CCL-81 cells.

Inhibiting viral entry is one mechanism by which chebulinic acid exerts its anti-HSV2 effects. To evaluate its impact on the entry of PEDV, a time-of-addition experiment was conducted. As illustrated in **Figures 6A, D**, the chebulinic-acid-treated group exhibited a significant difference between groups c and d for both YN13 and DR13-dORF3/GFP infection. It is well established that viral attachment and penetration predominantly occur within the first 2 h following PEDV infection (Wei et al., 2020). Therefore, these results suggested that chebulinic acid may influence either the attachment or penetration stages of PEDV. To confirm this, we assessed the effects of chebulinic acid on PEDV attachment and penetration, respectively. Chebulinic acid could inhibit both the attachment and penetration processes of YN13 (**Figures 6B, C**) and DR13-dORF3/GFP (**Figures 6E, F**) infection.

## 4 Discussion

PED is a significant threat to the global pig industry, as it causes severe and fatal diarrhea in piglets. Vaccination is considered as the most effective method to prevent PED. Nevertheless, because of the genetic variation of the PEDV, conventional vaccines are unable to offer sufficient protection for pigs against these evolving PEDV strains. Therefore, there is a critical need to develop effective treatments to combat PEDV infection.

Chebulinic acid is a significant tannin component derived from *T. chebula*, a traditional Chinese medicinal material that has been widely utilized in the treatment of respiratory and digestive diseases in both humans and animals. It has been reported to effectively inhibit the *in vitro* infection of several viruses responsible for serious diseases in humans. Li et al. demonstrated that chebulinic acid could inhibit the replication of the influenza A virus in MDCK cells by targeting viral neuraminidase (Li et al., 2020a). Furthermore, chebulinic acid has been shown to suppress the replication of HSV2 (Kesharwani et al., 2017), DENV, and CHIV (Thomas et al., 2022) by disrupting the processes of viral attachment and penetration. Notably, chebulinic acid was found to effectively inhibit the *in vitro* replication of SARS-CoV-2 and HCoV-OC43 (Ojha et al., 2023). However, the mechanisms by which chebulinic acid inhibits these two coronaviruses remain unclear. This study is the first to report the antiviral activity of chebulinic acid against PEDV, with evidence suggesting that inhibiting viral entry and the viral main protease play a crucial role in exerting this anti-PEDV effect.

PEDV strains can be categorized into two genotypes: GI (classical) and GII (variant) (Li et al., 2021). Currently, PEDV strains belonging to GII are the primary epidemic strains responsible for global PED outbreaks (Zhang et al., 2023). In this study, we assessed the effect of chebulinic acid on two PEDV strains. The DR13-dORF3/GFP strain is classified under GI, while the YN13 strain belongs to GII. Chebulinic acid exhibited a potent inhibitory effect on both DR13-dORF3/GFP and YN13 infections in CCL-81 cells, implying that it may have broad-spectrum anti-PEDV effects across both genotypes.

Since most non-structural proteins are directly or indirectly involved in the replication of the coronavirus genome, the cleavage of viral polyproteins by main protease represents a crucial step in the process of coronavirus infection. Furthermore, the cleavage of certain host proteins, such as the NF-κB essential modulator (*Nemo*) (Wang et al., 2015) and tetratricopeptide repeat protein 3 (*IFIT3*) (Zhu et al., 2017), by main protease is considered a significant mechanism through which the coronavirus counteracts host innate immunity. Thus, main protease has always been considered as an important drug target for anti-coronavirus research. Since the outbreak of SARS-CoV-2, the search for main protease inhibitors has remained a focal point in antiviral research. Different kinds of molecules, such as boceprevir, lopinavir, GC376, ensitrelvir, ebselen, and nirmatrelvir, have been reported as the inhibitors of SARS-COV2 main protease (Lu et al., 2021). In the case of PEDV, quercetin (Li et al., 2020b), baicalein, baicalin (Li et al., 2024), wogonin (Wang et al., 2023), and GC376 (Ye et al., 2020) were reported to inhibit PEDV infection targeting PEDV main protease. A docking study found that chebulinic acid could bind to the active site of SARS-CoV-2 main protease (Rudrapal et al., 2022). Speculating that chebulinic acid might engage with PEDV main protease, we discovered that this compound interacts with Gly144, His162, Pro188, and Gln191 within PEDV main protease through a docking analysis. Previous studies have indicated that the active sites of PEDV main protease include His41 and Cys144, while the S1 specificity pocket comprises Phe139, Ile140, Asn141, Gly142, Ala143, Cys144, His162, Gln163, and Glu165 (John et al., 2016; Ye et al., 2016). Consequently, it is plausible that chebulinic acid might associate with the active sites and the S1 specificity pocket of PEDV main protease, potentially leading to the inhibition of its activity. To evaluate the inhibitory effects of chebulinic acid on PEDV main protease, we conducted a FRET analysis. Since main protease, especially its active site and the binding pocket, is highly conserved among PEDV strains (John et al., 2016; Shamsi et al., 2022), only the main protease of PEDV YN13 strain was used in the FRET. The results of FRET showed that chebulinic acid effectively suppressed the activity of PEDV main protease. Although our research has demonstrated that chebulinic acid can bind to PEDV main protease and inhibit its function, the specific binding sites remain to be determined.

Virus entry represents the initial step in the viral life cycle and is a critical target for the development of antiviral therapies. For example, three clinically approved anti-HIV drugs—ibalizumab, maraviroc, and fostemsavir—function by inhibiting HIV entry into host cells through the disruption of interactions between viral receptor-binding proteins and their corresponding cellular receptors (Lu et al., 2021). Previous studies have shown that chebulinic acid can inhibit the entry of HSV2, DENV, and CHIV into their host cells (Kesharwani et al., 2017; Thomas et al., 2022). In line with these findings, our results indicate that chebulinic acid can also inhibit PEDV entry into CCL-81 cells. Furthermore, pre-treating PEDV with chebulinic acid enhanced its anti-PEDV effect, while pre-treating the cells did not. This suggested that chebulinic acid may inhibit PEDV entry by interacting with viral proteins rather than with the cellular surface proteins. For most enveloped viruses, the entry process consists of two key stages: attachment to and penetration into the host cells (Majdoul and Compton, 2021). Our results demonstrated that chebulinic acid could affect both the attachment and penetration processes of PEDV. Since the S protein plays a vital role in binding to the cellular receptor and facilitating viral fusion into the host cell, chebulinic acid might inhibit PEDV entry by interacting with the PEDV S protein. In addition, PEDV S protein can be divided into S1 and S2 subunits, with the former being responsible for attachment and the latter for penetration (Jung et al., 2020). Given that the S protein exhibits significant variability between GI and GII and chebulinic acid can inhibit the attachment and penetration of both the DR13-dORF3/GFP and YN13 strains, it appears that chebulinic acid may impede PEDV entry by interacting with the conserved domains within both the S1 and S2 subunits. However, the detailed interaction between them needs further investigation.

In conclusion, we have demonstrated that chebulinic acid could potently inhibit the propagation of PEDV *in vitro*. This effect is likely associated with its inhibitory action on PEDV main protease and its impact on viral entry. Consequently, this study offers theoretical support for anti-PEDV drug development.

## Data Availability

The datasets presented in this study can be found in online repositories. The names of the repository/repositories and accession number(s) can be found in the article/supplementary material.
